# Attributable mortality of ARDS among critically ill patients with sepsis: a multicenter, retrospective cohort study

**DOI:** 10.1186/s12890-024-02913-1

**Published:** 2024-03-04

**Authors:** Dong-Hui Wang, Hui-Miao Jia, Xi Zheng, Xiu-Ming Xi, Yue Zheng, Wen-Xiong Li

**Affiliations:** 1 Department of Surgical Intensive Care Unit, Beijing Chao-yang Hospital, Capital Medical University, 8 Gongren Tiyuchang Nanlu, Chaoyang District, 100020 Beijing, China; 2 Department of Critical Care Medicine, Fuxing Hospital, Capital Medical University, Beijing, China

**Keywords:** Sepsis, Acute respiratory distress syndrome, Attributable mortality, Propensity score matching

## Abstract

**Background:**

Both sepsis and acute respiratory distress syndrome (ARDS) are common severe diseases in the intensive care unit (ICU). There is no large-scale multicenter study to clarify the attributable mortality of ARDS among septic patients. This study aimed to evaluate the excess mortality of ARDS in critically ill patients with sepsis.

**Methods:**

The data were obtained from a multicenter, prospective cohort study in 18 Chinese ICUs between January 2014 and August 2015. The study population was septic patients after ICU admission. The patients were categorized into two groups: those who developed ARDS (ARDS group) within seven days following a sepsis diagnosis and those who did not develop ARDS (non-ARDS group). Applying propensity score matching (PSM), patients were matched 1:1 as ARDS and non-ARDS groups. Mortality attributed to ARDS was calculated. Subsequently, we conducted a survival analysis to estimate the impact of ARDS on mortality. The primary endpoint was 30-day mortality after sepsis diagnosis.

**Results:**

2323 septic patients were eligible, 67.8% developed ARDS. After PSM, 737 patients with ARDS were matched 1:1 with 737 non-ARDS patients. ARDS’s overall 30-day attributable mortality was 11.9% (95% CI 7.5–16.3%, *p* < 0.001). Subgroup analysis showed that the 30-day attributable mortality of mild, moderate, and severe ARDS was 10.5% (95% CI 4.0-16.8%, *p* < 0.001), 11.6% (95% CI 4.7–18.4%, *p* < 0.001) and 18.1% (95% CI 4.5–30.9%, *p* = 0.006), respectively. ARDS was an independent risk factor for 30-day mortality, with adjusted hazard ratios of 1.30 (95% CI 1.03–1.64, *p* = 0.027), 1.49 (95% CI 1.20–1.85, *p* < 0.001), and 1.95 (95% CI 1.51–2.52, *p* < 0.001) for mild, moderate, and severe ARDS, respectively.

**Conclusions:**

The overall 30-day attributable mortality of ARDS among critically ill patients with sepsis was 11.9%. Compared with mild and moderate ARDS, severe ARDS contributed more to death. ARDS was significantly associated with an increase in the 30-day mortality.

**Supplementary Information:**

The online version contains supplementary material available at 10.1186/s12890-024-02913-1.

## Background

Sepsis, a life-threatening illness caused by acute infection characterized by dysregulated host inflammatory response and multiple organ dysfunction, is a leading cause of preventable death in the intensive care unit (ICU) [1–3]. According to a worldwide multicenter epidemiological survey in 2017, there were approximately 48.9 million cases of sepsis and 11 million sepsis-related deaths worldwide, representing 19.7% of all global deaths [4]. From 2017 to 2019, there were 13.1% of sepsis-related deaths in China, and this proportion is still increasing [5]. Acute respiratory distress syndrome (ARDS) is a clinical syndrome of acute hypoxic respiratory failure caused by pulmonary inflammation rather than cardiogenic pulmonary edema [6]. According to a recent international multicenter prospective cohort study, ARDS may be present in up to 10% of patients in the ICU, and the mortality ranges from about 34.9% in mild cases to 46.1% in severe cases [7]. ARDS usually develops in patients with predisposing conditions that induce a systemic inflammatory response, and sepsis is the most common extrapulmonary cause of ARDS [7–9]. Sepsis-related ARDS is a grave condition with greater disease severity and mortality [10].

Due to the complex condition of septic patients in the ICU, it is difficult to determine the exact proportion of sepsis-related deaths caused by ARDS itself. The estimation of the impact of ARDS on mortality is of vital importance for the design of future clinical trials [11]. UP to now, no large-scale multicenter study has clarified the attributable mortality of ARDS among critically ill patients with sepsis. Thus, we performed a secondary data analysis from a large, prospective multicenter cohort study and applied propensity score matching (PSM) analysis to eliminate confounding factors, calculate attributable mortality of ARDS in critically ill patients with sepsis, and assess whether ARDS was an independent risk factor for 30-day mortality.

## Materials and methods

### Study setting and participants

Data for the PSM analysis were obtained from a prospective multicenter cohort study conducted by the China Critical Care Sepsis Trial (CCCST) working group in 18 Chinese ICUs. The period was from January 1, 2014, to August 31, 2015. The CCCST aimed to investigate the epidemiology and characteristics of critically ill patients with sepsis, which recruited admitted patients and stayed in the ICU for at least 24 h. Our study focused on patients with an unequivocal diagnosis of sepsis, meeting sepsis 3.0 criteria [1], after ICU admission. Patients were observed for the occurrence of ARDS within the first seven days after diagnosis of sepsis, as ARDS is characterized by the sudden onset or worsening development of breathing difficulty within one week of a clear incentive. The exclusion criteria included: (1) patients already suffered from ARDS of ICU admission; (2) ARDS occurred before sepsis; (3) ARDS occurred seven days later after the diagnosis of sepsis; (4) missing data; (5) patients younger than 18 years of age. All patients or their relatives had signed an informed consent form. The management of patients with sepsis and ARDS followed the International Guidelines for the Management of Severe Sepsis and Septic Shock (2012) [12].

The included patients were categorized into two groups: those who developed ARDS (ARDS group) within seven days following a sepsis diagnosis and those who did not develop ARDS (non-ARDS group).

### Definitions and clinical outcomes

The definition of sepsis 3.0 was used to define sepsis [1]. For cases before 2016, this definition was applied retrospectively. Septic shock was defined as sepsis requiring vasopressors after adequate fluid resuscitation to maintain mean arterial pressure (MAP) ≥ 65 mmHg and blood lactate concentration ≥ 2 mmol/L [1, 13]. ARDS was diagnosed according to the Berlin Definition (2012) [14]. At least two physicians independently assessed ARDS based on the clinical condition, chest X-ray or computed tomography scan, and arterial blood gases. The severity of ARDS was determined by oxygenation index according to the Berlin classification, using the oxygenation index at the time of the first diagnosis of ARDS, namely, mild (200 mmHg < PaO_2_/FiO_2_ ≤ 300 mmHg with PEEP or CPAP ≥ 5 cmH_2_O), moderate (100 mmHg < PaO_2_/FiO_2_ ≤ 200 mmHg with PEEP ≥ 5 cmH_2_O), and severe (PaO_2_/FiO_2_ ≤ 100 mmHg with PEEP ≥ 5 cmH_2_O).

The primary outcome was 30-day mortality after sepsis diagnosis. The secondary outcome was the length of stay (LOS) in ICU days, LOS in hospital days, ICU mortality, and hospital mortality.

### Data collection

Information collected from the dataset included demographic characteristics [gender, age, and body mass index (BMI)], comorbidities [chronic obstructive pulmonary disease (COPD) or asthma, cardiovascular disease, hypertension, diabetes, cancer, chronic kidney disease (CKD) and chronic liver disease], admission type, site of infection, organism responsible for sepsis, and diagnosis. The severity of the illness was represented by the Acute Physiology and Chronic Health Evaluation II (APACHE II) score and the Sequential Organ Failure Assessment (SOFA) score on the day of sepsis diagnosis. We collected clinical management data, including mechanical ventilation and renal replacement therapy (RRT), on the day of sepsis diagnosis. We recorded daily oxygenation index (PaO_2_/FiO_2_), positive end-expiratory pressure (PEEP), use of corticosteroid, mean arterial pressure (MAP), and the occurrence of septic shock and ARDS within seven days of the definitive diagnosis of sepsis. We also recorded the LOS in ICU days, LOS in hospital days, ICU mortality, 30-day mortality, and hospital mortality.

### Statistical analysis

**Fig. 1:**
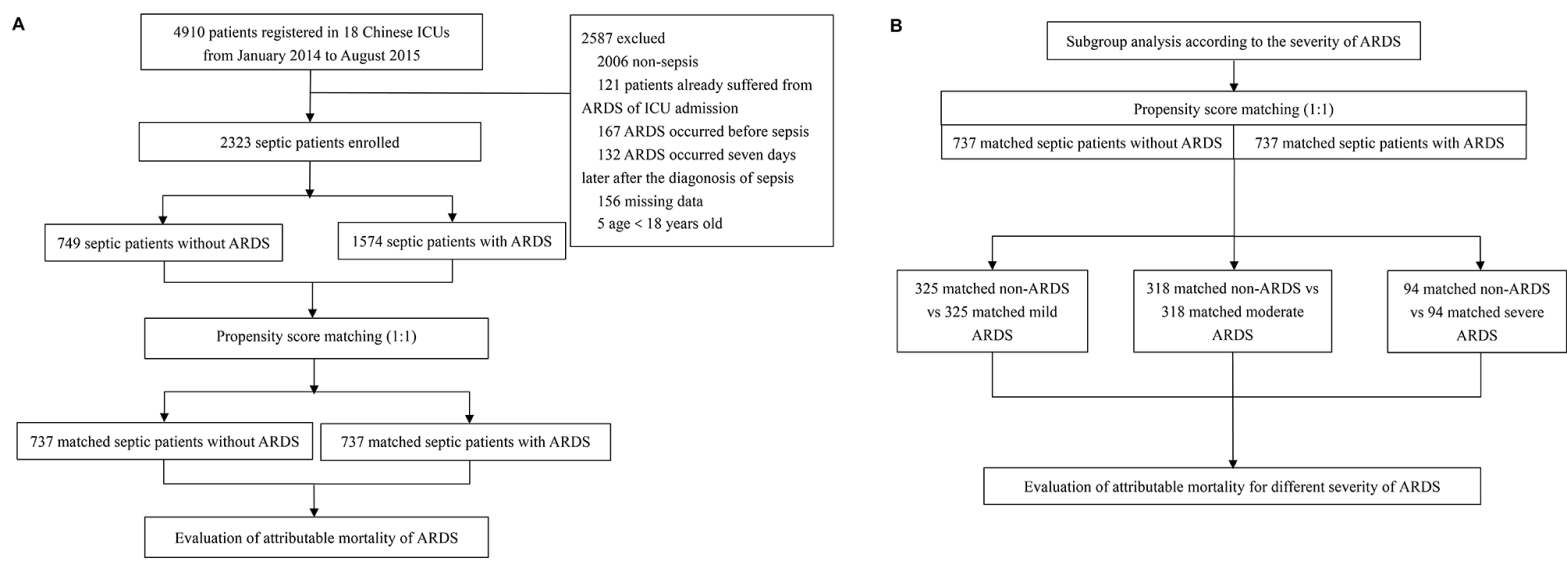
Study flow diagram. (**A**) Flowchart of all the participants. (**B**) Flowchart of the subgroup analysis. Abbreviations: ICU, intensive care unit; ARDS, acute respiratory distress syndrome

Statistical analysis was performed using SPSS statistics 26 (IBM, Chicago, IL) and R 4.2.3 (R Project for Statistical Computing). Continuous variables were presented as medians (interquartile range, IQR) and categorical variables as percentages. Continuous data between two groups were compared using the Mann-Whitney U test, and categorical variables were compared using the Chi-square test. Applying PSM between septic patients with and without ARDS to estimate the excess mortality attributed to ARDS (Fig. 1A). Using the “matchit” package for PSM can effectively eliminate the impact of baseline characteristics and clinical covariates on the outcome. PSM estimators included age, gender, BMI, comorbidities, APACHE II score, SOFA score, mechanical ventilation, RRT, MAP, and septic shock. Analysis was based on the lowest MAP. We established a matched control sample by using the nearest neighbor method. The value for the ratio was set to 1.0, and the width of the caliper was set to 0.09. The standardized differences were used to examine the covariate balance before and after matching. A value of 25% was considered a threshold for meaningful differences. McNemar’s test was applied to sensitivity analysis to assess the stability of outcomes [15]. Then, the 30-day mortality in matched septic patients with and without ARDS was calculated. Subgroup analysis of matched septic patients with and without ARDS was based on the severity of ARDS (Fig. 1B). Attributable mortality refers to the proportion of deaths that can be statistically attributed to the underlying cause [16], in this case, ARDS. The attributable mortality of ARDS was calculated by subtracting the mortality of matched septic patients without ARDS from the mortality of matched septic patients with ARDS. A 95% confidence interval (CI) for the attributable mortality difference was calculated by Newcombe’s method [17].

All ARDS and non-ARDS patients were included in the survival analysis. The Kaplan-Meier survival curve was used to describe the survival status of each group. The log-rank test was used to compare the survival time and survival rate differences between groups. The multivariate Cox proportional hazard model was used to calculate the hazard ratio (HR) of 30-day mortality and the corresponding 95% CI after adjusting for age, gender, BMI, comorbidities (COPD or asthma, cardiovascular disease, hypertension, diabetes, cancer, CKD and chronic liver disease), the need for mechanical ventilation and RRT, APACHE II score, SOFA score, septic shock, and MAP. Subsequently, the model was simplified by applying the stepwise method to derive the optimal model. All tests were two-tailed, and *p* < 0.05 was considered statistically significant.

## Result

### Characteristics of the demographics and proportion of ARDS

Of all 4910 patients in the study, 2323 septic patients were included in this analysis (Table 1), which included 749 (32.2%) cases of non-ARDS and 1574 (67.8%) cases of ARDS. The ARDS diagnosis time was shown in Supplemental Material Table S1. Of the total, 1490 (64.1%) were male. The median (IQR) age was 64 (50–77) years. Compared to the non-ARDS group, patients in the ARDS group had a higher BMI (*p* < 0.05). In total, 179 (7.7%) patients had COPD or asthma. The proportion of COPD or asthma was higher in the ARDS group (*p* < 0.05). 1311 (56.4%) were medical. The most common site of infection leading to sepsis was the lungs, accounting for 49.8%. The median (IQR) APACHE II score and SOFA score were 19 (14–25) and 7 (5–10) on the day of sepsis diagnosis, respectively. The ARDS group had higher APACHE II and SOFA scores (*p* < 0.05). 1809 (77.9%) and 439 (18.9%) patients received mechanical ventilation and RRT separately. 1133 (48.8%) patients suffered from septic shock. The ARDS group had a higher probability of receiving mechanical ventilation, RRT and experiencing septic shock (*p* < 0.05). In the entire cohort, the median (IQR) LOS in ICU and hospital were 8 (4–17) days and 18 (10–28) days, respectively. The 30-day mortality of ARDS was 33.1%.

**Table 1:** Baseline characteristics of septic patients classified by ARDS

Variables	All patients *n* = 2323	Non-ARDS group *n* = 749	ARDS group *n* = 1574	p-value
Male, n (%)	1490 (64.1)	450 (60.1)	1040 (66.1)	0.005
Age, median (IQR), years	64 (50–77)	62 (47–75)	65 (51–77)	< 0.001
BMI, median (IQR), kg/m^2^	23.3 (20.9–25.3)	22.9 (20.8–24.8)	23.4 (21.2–25.4)	0.002
Chronic comorbidities, n (%)				
COPD/asthma	179 (7.7)	32 (4.3)	147 (9.3)	< 0.001
Cardiovascular disease	362 (15.6)	122 (16.3)	240 (15.2)	0.518
Hypertension	775 (33.4)	238 (31.8)	537 (34.1)	0.263
Diabetes	449 (19.3)	149 (19.9)	300 (19.1)	0.634
Cancer	231 (9.9)	78 (10.4)	153 (9.7)	0.602
CKD	148 (6.4)	41 (5.5)	107 (6.8)	0.222
Chronic liver disease	43 (1.9)	9 (1.2)	34 (2.2)	0.109
Admission type, n (%)				
Medical	1311 (56.4)	429 (57.3)	882 (56.0)	< 0.001
Surgical	482 (20.7)	200 (26.7)	282 (17.9)	< 0.001
Emergency	530 (22.8)	120 (16.0)	410 (26.0)	< 0.001
Site of infection, n (%)				
Lung	1156 (49.8)	289 (38.6)	867 (55.1)	< 0.001
Abdomen	448 (19.3)	219 (29.2)	229 (14.5)	< 0.001
Bloodstream	345 (14.9)	112 (15.0)	233 (14.8)	0.924
Urinary tract	317 (13.6)	104 (13.9)	213 (13.5)	0.817
Others	57 (2.4)	25 (3.3)	32 (2.1)	0.057
Organism responsible for sepsis, n (%)				
GPC	471 (20.3)	143 (19.1)	328 (20.8)	0.328
GNB	548 (23.6)	173 (23.1)	375 (23.8)	0.700
Virus	106 (4.6)	31 (4.1)	75 (4.8)	0.499
Fungus	73 (3.1)	21 (2.8)	52 (3.3)	0.519
Others	40 (1.7)	11 (1.5)	29 (1.8)	0.517
PaO_2_/FiO_2_, median (IQR), mmHg	229 (151–350)	400 (355–434)	178 (122–232)	< 0.001
Use of corticosteroid, n (%)	396 (17.0)	114 (15.2)	282 (17.9)	0.106
APACHE II score, median (IQR)	19 (14–25)	17 (12–23)	20 (15–26)	< 0.001
SOFA score, median (IQR)	7 (5–10)	6 (3–9)	8 (5–11)	< 0.001
Mechanical ventilation, n (%)	1809 (77.9)	528 (70.5)	1281 (81.4)	< 0.001
RRT, n (%)	439 (18.9)	112 (15.0)	327 (20.8)	< 0.001
Septic shock, n (%)	1133 (48.8)	343 (45.8)	790 (50.2)	0.048
MAP, median (IQR), mmHg	75 (60–90)	73 (60–87)	76 (61–90)	0.095
Outcomes				
LOS in ICU, median (IQR), days	8 (4–17)	6 (3–15)	9 (5–18)	< 0.001
LOS in hospital, median (IQR), days	18 (10–28)	17 (9–28)	19 (11–29)	0.019
ICU mortality, n (%)	363 (15.6)	82 (10.9)	280 (17.8)	< 0.001
30-day mortality, n (%)	649 (27.9)	128 (17.1)	521 (33.1)	< 0.001
Hospital mortality, n (%)	875 (37.7)	178 (23.8)	697 (44.3)	< 0.001

Abbreviations: ARDS, acute respiratory distress syndrome; BMI, body mass index; COPD, chronic obstructive pulmonary disease; CKD, chronic kidney disease; GPC, Gram positive cocci; GNB, Gram negative bacteria; APACHE II, acute physiologic and chronic health evaluation II; SOFA, sequential organ failure assessment; RRT, renal replacement therapy; MAP, mean arterial pressure; LOS, length of stay; ICU, intensive care unit

Among 1,574 ARDS patients, 603 (38.3%) were mild, 707 (44.9%) were moderate, and 264 (16.8%) were severe. The 30-day mortality was 26.7%, 33.9%, and 45.5%, respectively (*p* < 0.05). Baseline characteristics of patients with ARDS by severity category were shown in Supplemental Material Table S2.

### Attributable mortality of ARDS in septic patients

**Fig. 2:**
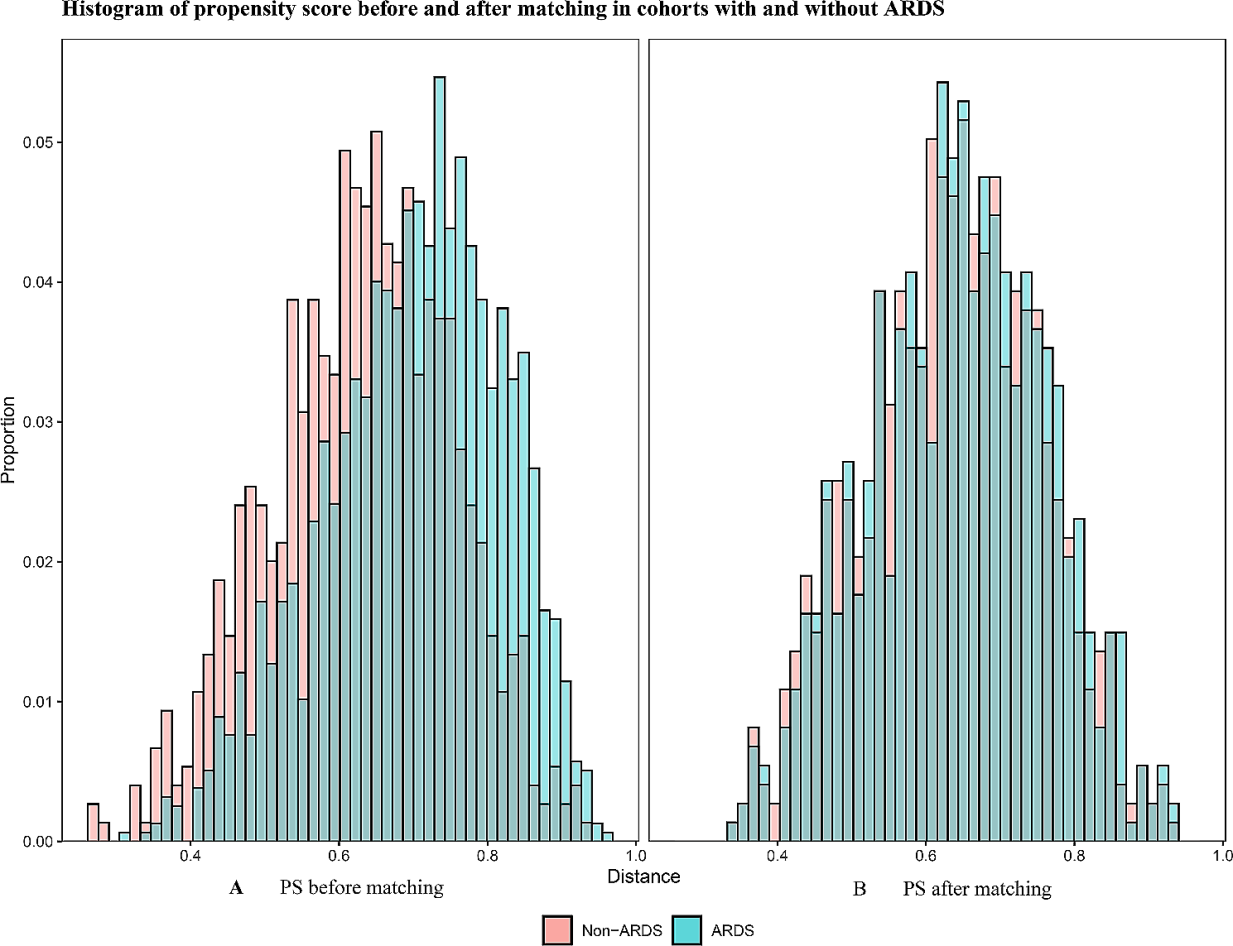
Histograms of propensity score before and after matching in cohorts with and without ARDS. (**A**) PS before matching. (**B**) PS after matching. Abbreviations: PS, propensity score

Of the 2323 septic patients, 737 patients with ARDS were matched 1:1 with 737 non-ARDS patients, according to PSM. Characteristics and standardized differences of the matched patients in the two groups were displayed in Table 2. The standardized differences before and after matching for all matched variables were less than 25%, meaning no variable exhibited a significant imbalance. The histograms of propensity score before and after matching in cohorts with and without ARDS were shown in Fig. 2. The density plot of propensity score before and after matching in cohorts with and without ARDS was shown in Supplemental Material Fig. S1. The Q-Q plots of the balance of the covariates were presented in Supplemental Material Fig. S2. The Jitter plots of the distribution of propensity scores were shown in Supplemental Material Fig. S3. The standardized difference before and after matching in cohorts with and without ARDS was shown in Supplemental Material Fig. S4. The 30-day mortality of the matched patients with total ARDS was 216 of 737 (29.3%) compared with 128 of 737 (17.4%) for their matched controls without ARDS (*p* < 0.001). The overall 30-day attributable mortality of total ARDS was 11.9% (95% CI 7.5–16.3%).

**Table 2:** Characteristics between matched septic patients with and without ARDS

Variables	Non-ARDS group *n* = 737	ARDS group *n* = 737	p- value	Standardized difference (%)
Male, n (%)	445 (60.4)	456 (61.9)	0.557	0.1
Age, median (IQR), years	62 (48–75)	64 (48–77)	0.204	0.1
BMI, median (IQR), kg/m^2^	22.9 (20.8–24.9)	22.9 (20.9–25.1)	0.686	2.5
Chronic comorbidities, n (%)				
COPD/asthma	32 (4.3)	33 (4.5)	0.899	5.0
Cardiovascular disease	121 (16.4)	128 (17.4)	0.627	4.6
Hypertension	237 (32.2)	241 (32.7)	0.824	2.3
Diabetes	149 (20.2)	145 (19.7)	0.794	2.1
Cancer	77 (10.5)	65 (8.8)	0.289	8.5
CKD	41 (5.6)	48 (6.5)	0.444	7.4
Chronic liver disease	9 (1.2)	7 (1.0)	0.817	6.4
APACHE II score, median (IQR)	17 (12–23)	18 (13–23)	0.090	10.5
SOFA score, median (IQR)	6 (3–9)	7 (4–9)	0.058	21.8
Mechanical ventilation, n (%)	527 (71.5)	539 (73.1)	0.485	2.3
RRT, n (%)	112 (15.2)	124 (16.8)	0.394	2.9
Septic shock, n (%)	336 (45.6)	340 (46.1)	0.834	12.6
MAP, median (IQR), mmHg	74 (60–88)	75 (62–88)	0.787	1.5

Abbreviations: ARDS, acute respiratory distress syndrome; BMI, body mass index; COPD, chronic obstructive pulmonary disease; CKD, chronic kidney disease; APACHE II, acute physiologic and chronic health evaluation II; SOFA, sequential organ failure assessment; RRT, renal replacement therapy; MAP, mean arterial pressure

**Fig. 3:**
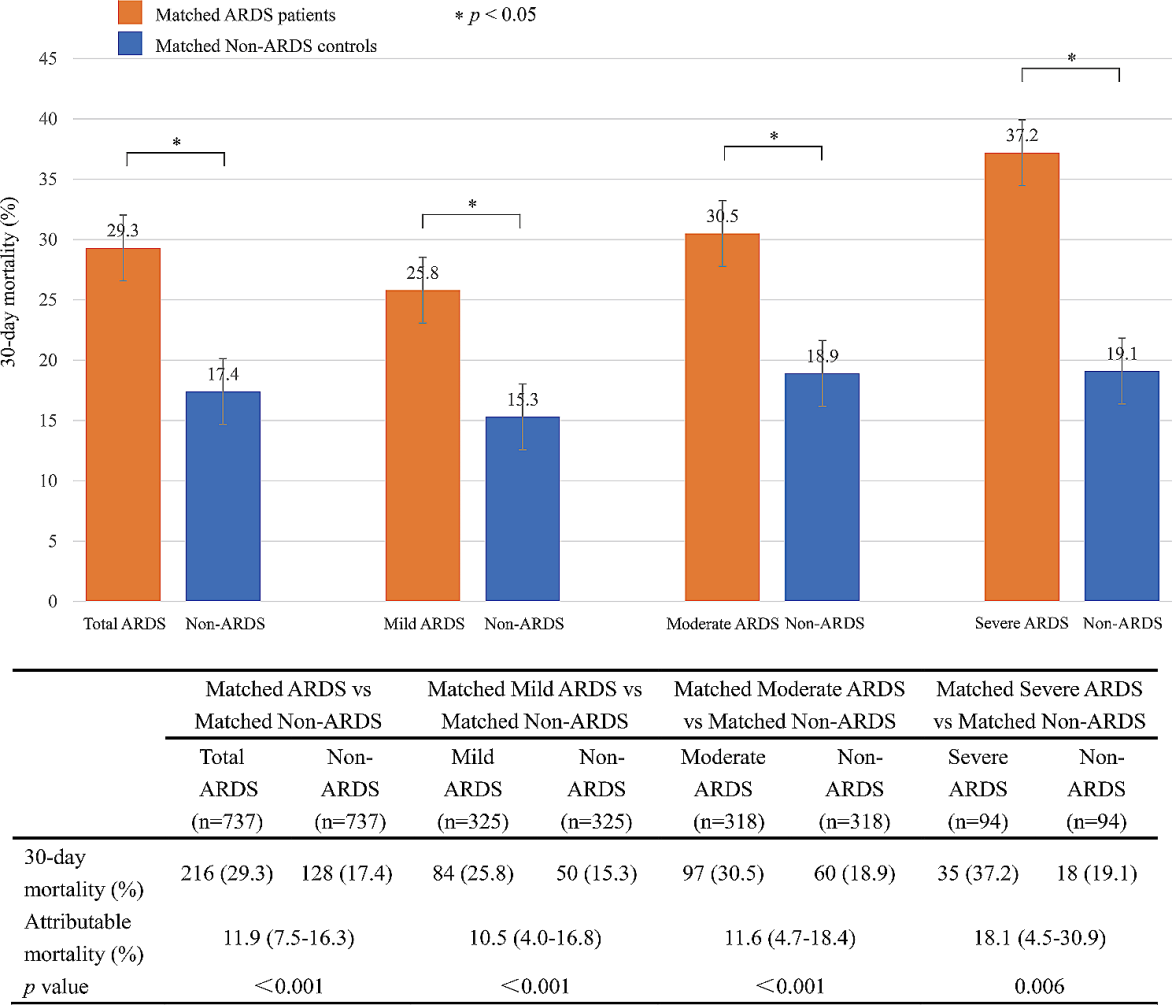
The attributable mortality of total ARDS and different severity of ARDS in subgroups. Abbreviations: ARDS, acute respiratory distress syndrome

Matched septic patients with and without ARDS were subgrouped according to ARDS severity. A total of 325 patients with mild ARDS, 318 patients with moderate ARDS, and 94 patients with severe ARDS were matched 1:1 with their controls without ARDS, respectively. Subgroup analysis showed that the 30-day attributable mortality of mild, moderate, and severe ARDS was 10.5% (95% CI 4.0-16.8%, *p* < 0.001), 11.6% (95% CI 4.7–18.4%, *p* < 0.001) and 18.1% (95% CI 4.5–30.9%, *p* = 0.006), respectively, as shown in Fig. 3.

### Sensitivity analysis

Propensity score analysis was based on the assumption that all relevant covariates had been measured. Therefore, we could disregard the effect of unmeasured covariates. We conducted a sensitivity analysis to assess the impact of unmeasured covariates on the results. The gamma value reflects the sensitivity of the estimated treatment effect to the hidden bias of the unmeasured covariates [18, 19]. The effect estimate was considered robust to hidden bias if the statistical significance did not change until a very large gamma. Our sensitivity analysis showed that the estimation of 30-day attributable mortality of ARDS was reliable for the hidden bias of unmeasured covariates, with a gamma coefficient of up to 3. The sensitivity analysis of 30-day attributable mortality results was shown in Supplemental Material Table S3.

### Survival analysis

**Fig. 4:**
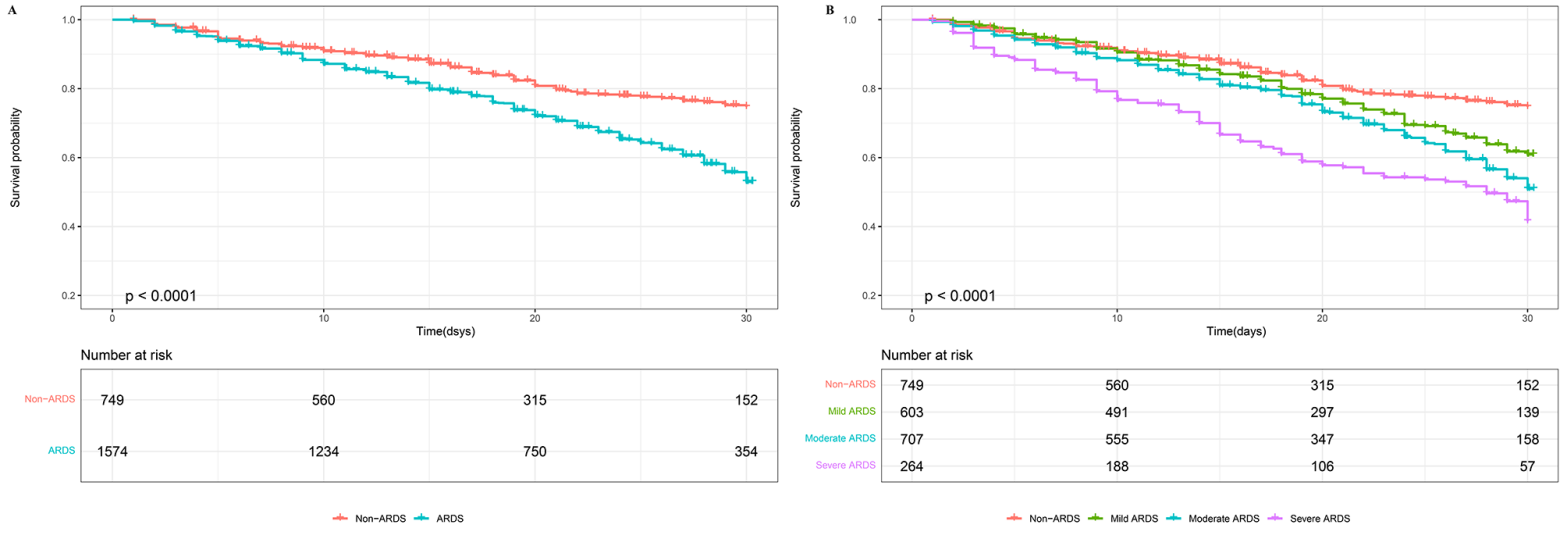
Kaplan-Meier survival analysis. (**A**) Kaplan-Meier survival analysis comparing non-ARDS and ARDS groups. (**B**) Kaplan-Meier survival analysis comparing non-ARDS, mild ARDS, moderate ARDS, and severe ARDS groups. *p* < 0.001 for all comparisons. The numbers of patients at risk at each time point were shown below the graph. Abbreviations: ARDS, acute respiratory distress syndrome

In Fig. 4A, the Kaplan-Meier curve revealed significantly higher mortality in the ARDS group compared to the non-ARDS group (*p* < 0.001). Survival probabilities at the 30-day were 74.7% (95% CI 70.6–79.0%) for non-ARDS patients and 53.0% (95% CI 49.8–56.3%) for ARDS patients. Subgroup analysis showed a statistically significant decrease in survival with worsening ARDS severity (*p* < 0.001), as shown in Fig. 4B. Survival probabilities at the 30-day were 60.9% (95% CI 56.0-66.3%) for mild ARDS patients, 50.9% (95% CI 46.3–56.0%) for moderate ARDS patients, and 41.5% (95% CI 34.7–49.7%) for severe ARDS patients.

**Fig. 5:**
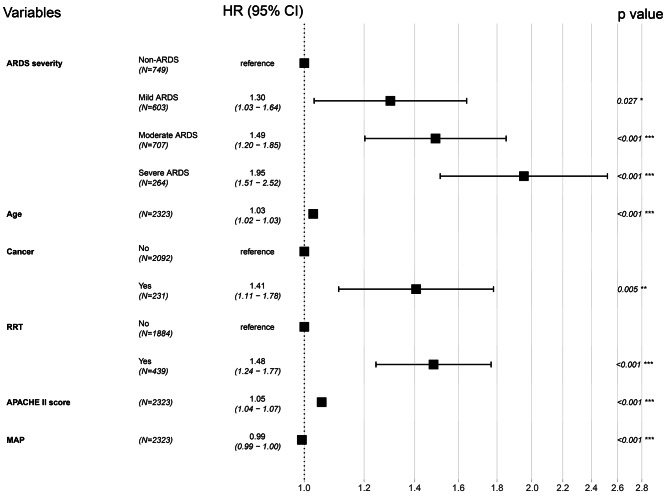
Forest plot of multivariable Cox proportional hazard regression analysis for 30-day mortality stratified by severity of ARDS. Abbreviations: ARDS, acute respiratory distress syndrome; RRT, renal replacement therapy; APACHE II, acute physiologic and chronic health evaluation II; MAP, mean arterial pressure

In our optimal multivariate Cox regression adjusted model (Fig. 5), ARDS was associated with higher 30-day mortality. In the adjusted model, mild, moderate, and severe ARDS were independent risk factors of 30-day mortality, with adjusted HRs of 1.30 (95% CI 1.03–1.64, *p* = 0.027), 1.49 (95% CI 1.20–1.85, *p* < 0.001), and 1.95 (95% CI 1.51–2.52, *p* < 0.001), respectively. Supplemental Material Table S4 showed the optimal multivariable Cox proportional hazard regression analysis for 30-day mortality stratified by severity of ARDS. Supplemental Material Table S5 and Fig. S5 showed the full model of the multivariable Cox regression.

## Discussion

Sepsis is a major global health issue that affects millions of people worldwide every year and leads to one-third to one-sixth of deaths [20–23]. Cytokine storms during sepsis can lead to intractable lung inflammation and the development of ARDS, causing lung damage and high mortality [24]. ARDS is a common clinical complication of sepsis. It is associated with increased mortality and a threatening impact on human health. Compared with ARDS due to other causes, sepsis-associated ARDS has a higher mortality rate of 30–40% [25]. Sepsis is a risk factor and a leading cause of death in ARDS, with a close relationship and mutual influence. Due to the complex condition of critically ill patients, it is hard to identify the cause of death in a short time. We determined the proportion of deaths attributable to ARDS in septic patients. This finding would help calculate the sample size for future clinical trials of ARDS in critically ill patients with sepsis. Our research found that the overall 30-day attributable mortality of ARDS in septic patients was 11.9%. Subgroup analysis showed that the excess 30-day mortality of mild, moderate, and severe ARDS were 10.5%, 11.6%, and 18.1%, respectively. Severe ARDS was associated with higher excess mortality than mild and moderate ARDS, highlighting the importance of early intervention to prevent the progression of ARDS.

The studies on the attributable mortality of ARDS vary significantly among different studies. In 2020, a study by Auriemma [26] concluded that the proportion of ARDS in-hospital attributable mortality risk was between 27% and 37% in septic patients through a retrospective analysis of two prospective sepsis cohorts studies in ICU, with an attributable mortality of 27% in the Early Assessment of Renal and Lung Injury (EARLI) [27, 28] cohort including 474 patients, and 37% in the Validating Acute Lung Injury markers for Diagnosis (VALID) [29, 30] cohort including 337 patients. Their results were higher than ours, probably because of the relatively higher severity of the disease in their study population, and their primary outcome was in-hospital mortality. Shortly after the publication of Auriemma’s research [26], de Grooth [31] penned a correspondence to the editor, outlining potential reasons for the overstatement of the ARDS-related mortality risk proportion. The analysis in Auriemma’s study may not have accounted for certain extrapulmonary organ failures, which could have elevated the estimated ARDS attributable risk. In another study, Torres [32] found that ARDS increased 28-day mortality by 15% in critically ill patients by analyzing 658 septic patients with significant increases in mortality for severe ARDS (23%) and moderate ARDS (16%), which was similar to our findings. However, they found that mild ARDS did not increase mortality. Our study suggested that mild ARDS also increases mortality by 10.5%, which was inconsistent with their results, possibly due to the differences in the study population. However, the sample sizes of the two studies conducted respectively by Auriemma and Torres were relatively small. Recently, through the secondary analysis of data from the Large observational study to UNderstand the Global impact of Severe Acute respiratory FailurE (LUNG SAFE) [7] cohort, Saha reported that the average overall attributable mortality of ARDS was between 20.9% and 38.0% [33]. Their research results were higher than ours, probably because the ARDS population they included not only had sepsis but also other causes, such as trauma, blood transfusion, and drowning.

We found that septic patients with ARDS had higher ICU stay, hospital stay, ICU mortality, hospital mortality, and 30-day mortality than non-ARDS patients (*p* < 0.05). In our study, the ARDS 30-day mortality in septic patients was 33.1%. Upon further analysis, the 30-day mortality of ARDS was 26.7% for mild ARDS patients, 33.9% for moderate ARDS patients, and 45.5% for severe ARDS patients, similar to the LUNG SAFE study in 2016. Our survival analysis showed that as the severity of ARDS worsens, mortality tends to increase. We also found that ARDS was associated with higher 30-day mortality, highlighting ARDS as an independent risk factor for 30-day mortality. The 30-day mortality of mild, moderate, and severe ARDS patients was 0.30 times, 0.49 times, and 0.95 times higher than that of non-ARDS patients, respectively, indicating a worse prognosis for patients with severe ARDS.

This study has the following strengths. The data came from a large, multicenter, prospective clinical study in China. Secondly, we used the definition of sepsis 3.0 and the Berlin criteria for ARDS. Thirdly, the new statistical PSM method was used to eliminate the confounding factors, similar to the randomized controlled trial, and sensitivity analysis was carried out. However, there were also a few limitations. Firstly, it was a retrospective secondary analysis that might introduce unmeasured bias. Secondly, the site of infection was not incorporated into the propensity score analysis, as the model could not accommodate multi-categorical variables. Nevertheless, we extensively analyzed how unmeasured variables might affect the robustness of our propensity score analysis results. The sensitivity analysis indicated that excluding these variables did not markedly influence the stability of our findings. Thirdly, while around 39% of the patients in the non-ARDS group presented with sepsis of pulmonary origin, they did not meet the criteria for an ARDS diagnosis based on the PaO_2_/FiO_2_ ratio (greater than 300 mmHg) as defined in the Berlin definition acquired within the first seven days following sepsis diagnosis. Nonetheless, it is important to note that some patients could go on to develop ARDS more than seven days after being diagnosed with sepsis. Fourthly, the database only recorded the PEEP and PaO_2_/FiO_2_ ratio for the ventilation parameter, while other parameters were not recorded. The risk of ventilator-induced lung injury may increase the 30-day mortality in septic patients who developed ARDS, and this could be a potential cause of ARDS-related mortality in these patients. Notably, 16.8% of patients with moderate ARDS and 12.1% with severe ARDS did not undergo invasive mechanical ventilation in our study. The majority of these patients received non-invasive ventilation (NIV) with PEEP ≥ 5 cmH_2_O, while a smaller fraction underwent high-flow nasal cannula oxygen therapy (HFNC). HFNC can generate a flow-dependent positive airway pressure [34], which mimics the physiological effects of PEEP. However, the level of PEEP remained uncertain. In our study, we incorporated patients who received HFNC with PaO_2_/FiO_2_ ≤ 300 mmHg into the diagnosis and stratification of ARDS. These patients likely belonged to the ARDS category, as mentioned in the 2023 updated global definition of ARDS [35]. We note that confidence in the study’s findings may be compromised because HFNC did not have the PEEP parameter, which did not meet the Berlin definition. Additionally, when subgroup analysis was conducted according to the severity of ARDS, the relatively small amount of data for each subgroup resulted in a wide range of CIs for attributable mortality of ARDS. Thus, the subgroup analysis result should be interpreted carefully and further demonstrated in subsequent studies.

## Conclusion

The overall 30-day attributable mortality of ARDS among critically ill patients with sepsis was 11.9%. Compared with mild and moderate ARDS, severe ARDS contributed more to death. ARDS was significantly associated with an increase in the 30-day mortality.

### Electronic supplementary material

Below is the link to the electronic supplementary material.

Supplementary Material 1

## Data Availability

The datasets analyzed during the current study are available from the corresponding author upon reasonable request.

## Abbreviations

ARDS: Acute respiratory distress syndrome
ICU: Intensive care unit
PSM: Propensity score matching
BMI: Body mass index
COPD: Chronic obstructive pulmonary disease
CKD: Chronic kidney disease
APACHE II: Acute physiologic and chronic health evaluation II
SOFA: Sequential organ failure assessment
RRT: Renal replacement therapy
MAP: Mean arterial pressure
LOS: Length of stay
IQR: Interquartile range
HR: Hazard ratio
CI: Confidence interval
PEEP: Positive end-expiratory pressure
GPC: Gram positive cocci
GNB: Gram negative bacteria
CCCST: China Critical Care Sepsis Trial
HFNC: High-flow nasal cannula oxygen therapy
NIV: Non-invasive ventilation

## References

[1] Singer M, Deutschman CS, Seymour CW (2016). The Third International Consensus definitions for Sepsis and septic shock (Sepsis-3). JAMA.

[2] Cecconi M, Evans L, Levy M (2018). Sepsis and septic shock. Lancet.

[3] Horak J, Martinkova V, Radej J (2019). Back to basics: Recognition of Sepsis with New Definition. J Clin Med.

[4] Rudd KE, Johnson SC, Agesa KM (2020). Global, regional, and national sepsis incidence and mortality, 1990–2017: analysis for the global burden of Disease Study. Lancet.

[5] Weng L, Xu Y, Yin P (2023). National incidence and mortality of hospitalized sepsis in China. Crit Care.

[6] Meyer NJ, Gattinoni L, Calfee CS (2021). Acute respiratory distress syndrome. Lancet.

[7] Bellani G, Laffey JG, Pham T (2016). Epidemiology, patterns of Care, and mortality for patients with Acute Respiratory Distress Syndrome in Intensive Care Units in 50 countries. JAMA.

[8] Wheeler AP, Bernard GR (2007). Acute lung injury and the acute respiratory distress syndrome: a clinical review. Lancet.

[9] Bos LDJ, Ware LB (2022). Acute respiratory distress syndrome: causes, pathophysiology, and phenotypes. Lancet.

[10] Sheu CC, Gong MN, Zhai R (2010). Clinical characteristics and outcomes of sepsis-related vs non-sepsis-related ARDS. Chest.

[11] Shankar-Hari M, Harrison DA, Rowan KM (2018). Estimating attributable fraction of mortality from sepsis to inform clinical trials. J Crit Care.

[12] Dellinger RP, Levy MM, Rhodes A (2013). Surviving sepsis campaign: international guidelines for management of severe sepsis and septic shock: 2012. Crit Care Med.

[13] Kamath S, Hammad Altaq H, Abdo T (2023). Management of Sepsis and septic shock: what have we learned in the last two decades?. Microorganisms.

[14] Ranieri VM, Rubenfeld GD, Thompson BT (2012). Acute respiratory distress syndrome: the Berlin definition. JAMA.

[15] Lee J, Little TD (2017). A practical guide to propensity score analysis for applied clinical research. Behav Res Ther.

[16] Wacholder S, Benichou J, Heineman EF (1994). Attributable risk: advantages of a broad definition of exposure. Am J Epidemiol.

[17] Newcombe RG (1998). Improved confidence intervals for the difference between binomial proportions based on paired data. Stat Med.

[18] Rosenbaum PR (1991). Sensitivity analysis for matched case-control studies. Biometrics.

[19] Rosenbaum PR (1991). Discussing hidden bias in observational studies. Ann Intern Med.

[20] Evans L, Rhodes A, Alhazzani W (2021). Surviving Sepsis Campaign: International guidelines for Management of Sepsis and Septic Shock 2021. Crit Care Med.

[21] Fleischmann C, Scherag A, Adhikari NK (2016). Assessment of Global Incidence and Mortality of Hospital-treated Sepsis. Current estimates and limitations. Am J Respir Crit Care Med.

[22] Fleischmann-Struzek C, Mellhammar L, Rose N (2020). Incidence and mortality of hospital- and ICU-treated sepsis: results from an updated and expanded systematic review and meta-analysis. Intensive Care Med.

[23] Rhee C, Dantes R, Epstein L (2017). Incidence and Trends of Sepsis in US hospitals using clinical vs Claims Data, 2009–2014. JAMA.

[24] Li W, Li D, Chen Y (2022). Classic Signaling pathways in Alveolar Injury and Repair involved in Sepsis-Induced ALI/ARDS: New Research Progress and Prospect. Dis Markers.

[25] Stapleton RD, Wang BM, Hudson LD (2005). Causes and timing of death in patients with ARDS. Chest.

[26] Auriemma CL, Zhuo H, Delucchi K (2020). Acute respiratory distress syndrome-attributable mortality in critically ill patients with sepsis. Intensive Care Med.

[27] Agrawal A, Matthay MA, Kangelaris KN (2013). Plasma angiopoietin-2 predicts the onset of acute lung injury in critically ill patients. Am J Respir Crit Care Med.

[28] Kangelaris KN, Prakash A, Liu KD (2015). Increased expression of neutrophil-related genes in patients with early sepsis-induced ARDS. Am J Physiol Lung Cell Mol Physiol.

[29] O’Neal HR, Koyama T, Koehler EA (2011). Prehospital statin and aspirin use and the prevalence of severe sepsis and acute lung injury/acute respiratory distress syndrome. Crit Care Med.

[30] Russell DW, Janz DR, Emerson WL (2017). Early exposure to hyperoxia and mortality in critically ill patients with severe traumatic injuries. BMC Pulm Med.

[31] de Grooth HJ, Tuinman PR, Girbes ARJ (2020). The attributable mortality of acute respiratory distress syndrome. Intensive Care Med.

[32] Torres LK, Hoffman KL, Oromendia C (2021). Attributable mortality of acute respiratory distress syndrome: a systematic review, meta-analysis and survival analysis using targeted minimum loss-based estimation. Thorax.

[33] Saha R, Pham T, Sinha P (2023). Estimating the attributable fraction of mortality from acute respiratory distress syndrome to inform enrichment in future randomised clinical trials. Thorax.

[34] Papazian L, Corley A, Hess D (2016). Use of high-flow nasal cannula oxygenation in ICU adults: a narrative review. Intensive Care Med.

[35] Matthay MA, Arabi Y, Arroliga AC (2024). A new global definition of acute respiratory distress syndrome. Am J Respir Crit Care Med.

