# Supplementary material for: Attributable mortality of ARDS among critically ill patients with sepsis: a multicenter, retrospective cohort study
Source: BMC Pulm Med. 2024 Mar 4;24:110. doi: 10.1186/s12890-024-02913-1 (PMC10913263; doi:10.1186/s12890-024-02913-1)
Supplement: Supplementary file 1 — Supplementary Material 1 [file 12890_2024_2913_MOESM1_ESM.docx]

**Attributable mortality of ARDS among critically ill patients with sepsis: a multicenter, retrospective cohort study**

**Dong-Hui Wang^1^, Hui-Miao Jia^1^, Xi Zheng^1^, Xiu-Ming Xi^2^, Yue Zheng^1*^, Wen-Xiong Li^1*^**

^1^Department of Surgical Intensive Care Unit, Beijing Chao-yang Hospital, Capital Medical University, Beijing 100020, China

^2^Department of Critical Care Medicine, Fuxing Hospital, Capital Medical University, Beijing, China

**Supplemental Content**

**Supplemental Material Contents (in order of appearance in manuscript)**:

Supplemental Material Table S1. The ARDS diagnosis time in the ARDS group after diagnosis of sepsis

Supplemental Material Table S2. Baseline characteristics of patients with ARDS by severity category

Supplemental Material Fig. S1. Density plot of propensity score before and after matching in cohorts with and without ARDS

Supplemental Material Fig. S2. The Q-Q plots of the balance of the covariates

Supplemental Material Fig. S3. The Jitter plots of the distribution of propensity scores

Supplemental Material Fig. S4. The standardized difference before and after matching in cohorts with and without ARDS

Supplemental Material Table S3. Results of the sensitivity analysis of 30-day attributable mortality

Supplemental Material Table S4. The optimal multivariable Cox proportional hazard regression analysis for 30-day mortality stratified by severity of ARDS

Supplemental Material Table S5. The full model of the multivariable Cox regression

Supplemental Material Fig. S5. The forest plot for full model of the multivariable Cox regression

**Supplemental Material Table S1. The ARDS diagnosis time in the ARDS group after diagnosis of sepsis**

| **Variables** | **ARDS diagnosis time** | | | | | | |
| --- | --- | --- | --- | --- | --- | --- | --- |
|  | Day 1 | Day 2 | Day 3 | Day 4 | Day 5 | Day 6 | Day 7 |
| Number of patients with ARDS, n (%) | 207 (13.1) | 386 (24.5) | 471 (29.9) | 182 (11.6) | 113 (7.2) | 127 (8.1) | 88 (5.6) |

**Supplemental Material Table S2. Baseline characteristics of patients with ARDS by severity category**

| **Variables** | **Mild ARDS**  **n=603** | **Moderate ARDS**  **n=707** | **Severe ARDS**  **n=264** | ***p*-value** |
| --- | --- | --- | --- | --- |
| Male, n (%) | 392 (65.0) | 470 (66.5) | 178 (67.4) | 0.037 |
| Age, median (IQR), years | 64 (49-77) | 67 (53-79) | 64 (49-74) | 0.004 |
| BMI, median (IQR), kg/m^2^ | 23.4 (21.2-25.4) | 23.4 (21.3-25.4) | 23.4 (21.2-25.5) | 0.800 |
| Chronic comorbidities, n (%) | | | | |
| COPD/asthma | 43 (7.1) | 89 (12.6) | 15 (5.7) | <0.001 |
| Cardiovascular disease | 85 (14.1) | 121 (17.1) | 34 (12.9) | 0.259 |
| Hypertension | 200 (33.2) | 252 (35.6) | 85 (32.2) | 0.445 |
| Diabetes | 113 (18.7) | 138 (19.5) | 49 (18.6) | 0.940 |
| Cancer | 57 (9.5) | 65 (9.2) | 31 (11.7) | 0.627 |
| CKD | 40 (6.6) | 43 (6.1) | 24 (9.1) | 0.217 |
| Chronic liver disease | 13 (2.2) | 15 (2.1) | 6 (2.3) | 0.459 |
| Admission type, n (%) | | | | |
| Medical | 317 (52.6) | 389 (55.0) | 176 (66.7) | <0.001 |
| Surgical | 148 (24.5) | 105 (14.9) | 29 (11.0) | <0.001 |
| Emergency | 138 (22.9) | 213 (30.1) | 59 (22.3) | <0.001 |
| Site of infection, n (%) | | | | |
| Lung | 347 (57.5) | 383 (54.2) | 137 (51.9) | 0.247 |
| Abdomen | 75 (12.4) | 113 (16.0) | 41 (15.5) | 0.171 |
| Bloodstream | 77 (12.8) | 114 (16.1) | 42 (15.9) | 0.201 |
| Urinary tract | 69 (11.4) | 104 (14.7) | 40 (15.2) | 0.159 |
| Others | 13 (2.2) | 11 (1.6) | 8 (3.0) | 0.338 |
| APACHE II score, median (IQR) | 18 (13-24) | 20 (15-25) | 24 (18-29) | <0.001 |
| SOFA score, median (IQR) | 7 (4-9) | 8 (6-11) | 10 (7-13) | <0.001 |
| Mechanical ventilation, n (%) | 461 (76.5) | 588 (83.2) | 232 (87.9) | <0.001 |
| PEEP, median (IQR), cmH_2_O | 6 (5-7) | 8 (8-10) | 15 (12-18) | <0.001 |
| PaO_2_/FiO_2_, median (IQR), mmHg | 249 (221-270) | 156 (130-179) | 80 (63-91) | <0.001 |
| RRT, n (%) | 111 (18.4) | 146 (20.7) | 70 (26.5) | <0.001 |
| Septic shock, n (%) | 250 (41.5) | 374 (52.9) | 166 (62.9) | <0.001 |
| MAP, median (IQR), mmHg | 76 (63-93) | 77 (61-91) | 69 (58-86) | <0.001 |
| Outcomes | | | | |
| LOS in ICU, median (IQR), days | 9 (5-18) | 10 (5-19) | 9 (4-17) | 0.115 |
| LOS in hospital, median (IQR), days | 19 (11-29) | 19 (11-28) | 16 (9-28) | 0.028 |
| ICU mortality, n (%) | 90 (14.9) | 114 (16.1) | 76 (28.8) | <0.001 |
| 30-day mortality, n (%) | 161 (26.7) | 240 (33.9) | 120 (45.5) | <0.001 |
| Hospital mortality, n (%) | 239 (39.6) | 310 (43.8) | 148 (56.1) | <0.001 |

Abbreviations: ARDS, acute respiratory distress syndrome; BMI, body mass index; COPD, chronic obstructive pulmonary disease; CKD, chronic kidney disease; APACHE II, acute physiologic andchronic health evaluation II; SOFA, sequential organ failure assessment; PEEP, positive end-expiratory pressure; RRT, renalreplacement therapy; MAP, mean arterial pressure; LOS, length of stay; ICU, intensive care unit


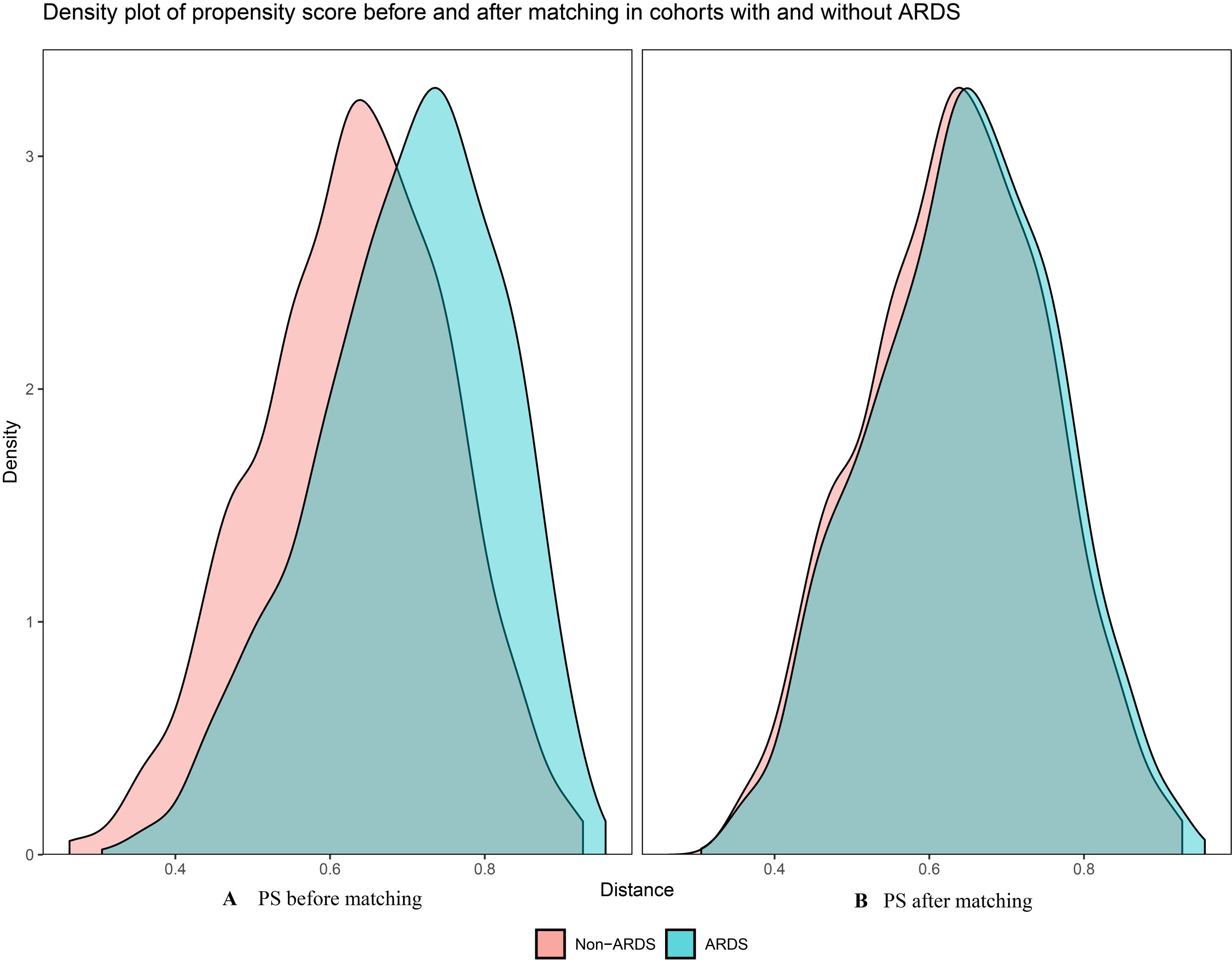


**Supplemental Material Fig. S1. Density plot of propensity score before and after matching in cohorts with and without ARDS** (**A**) PS before matching. (**B**) PS after matching. Abbreviations: PS, propensity score


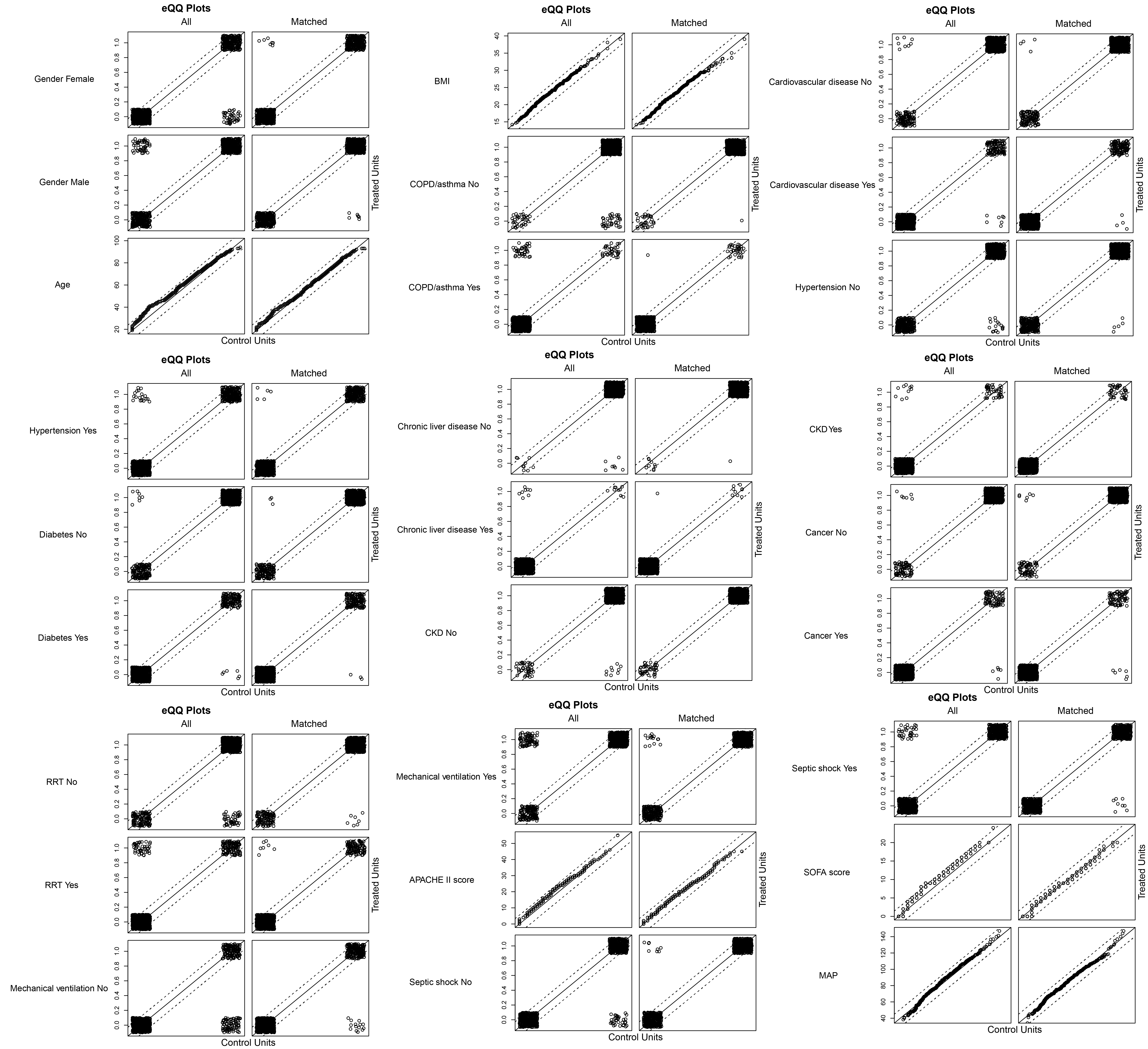


**Supplemental Material Fig. S2. The Q-Q plots of the balance of the covariates**

Abbreviations: ARDS, acute respiratory distress syndrome; BMI, body mass index; COPD, chronic obstructive pulmonary disease; CKD, chronic kidney disease; APACHE II, acute physiologic and chronic health evaluation II; SOFA, sequential organ failure assessment; RRT, renal replacement therapy; MAP, mean arterial pressure; LOS, length of stay; ICU, intensive care unit


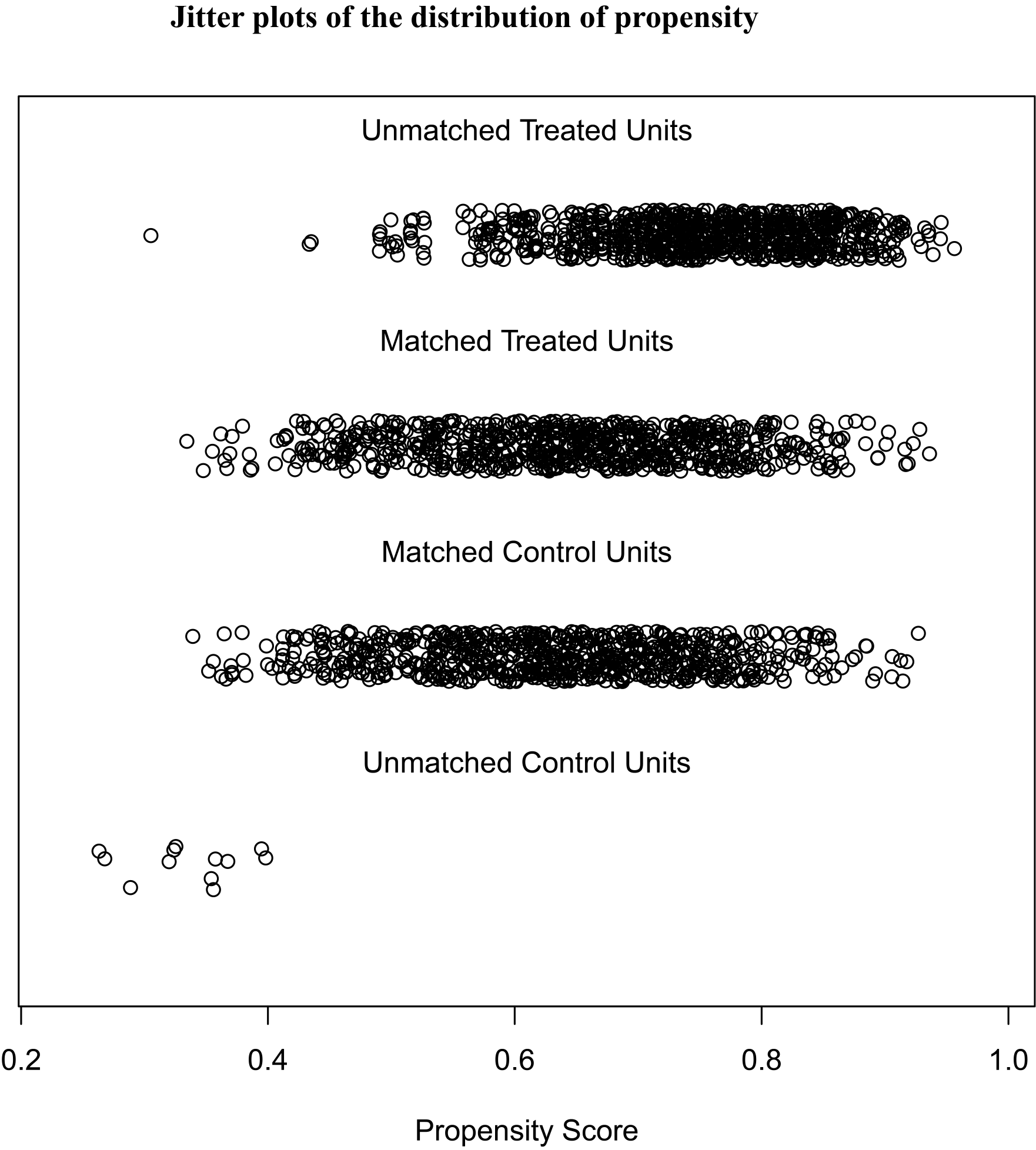


**Supplemental Material Fig. S3. The Jitter plots of the distribution of propensity scores**


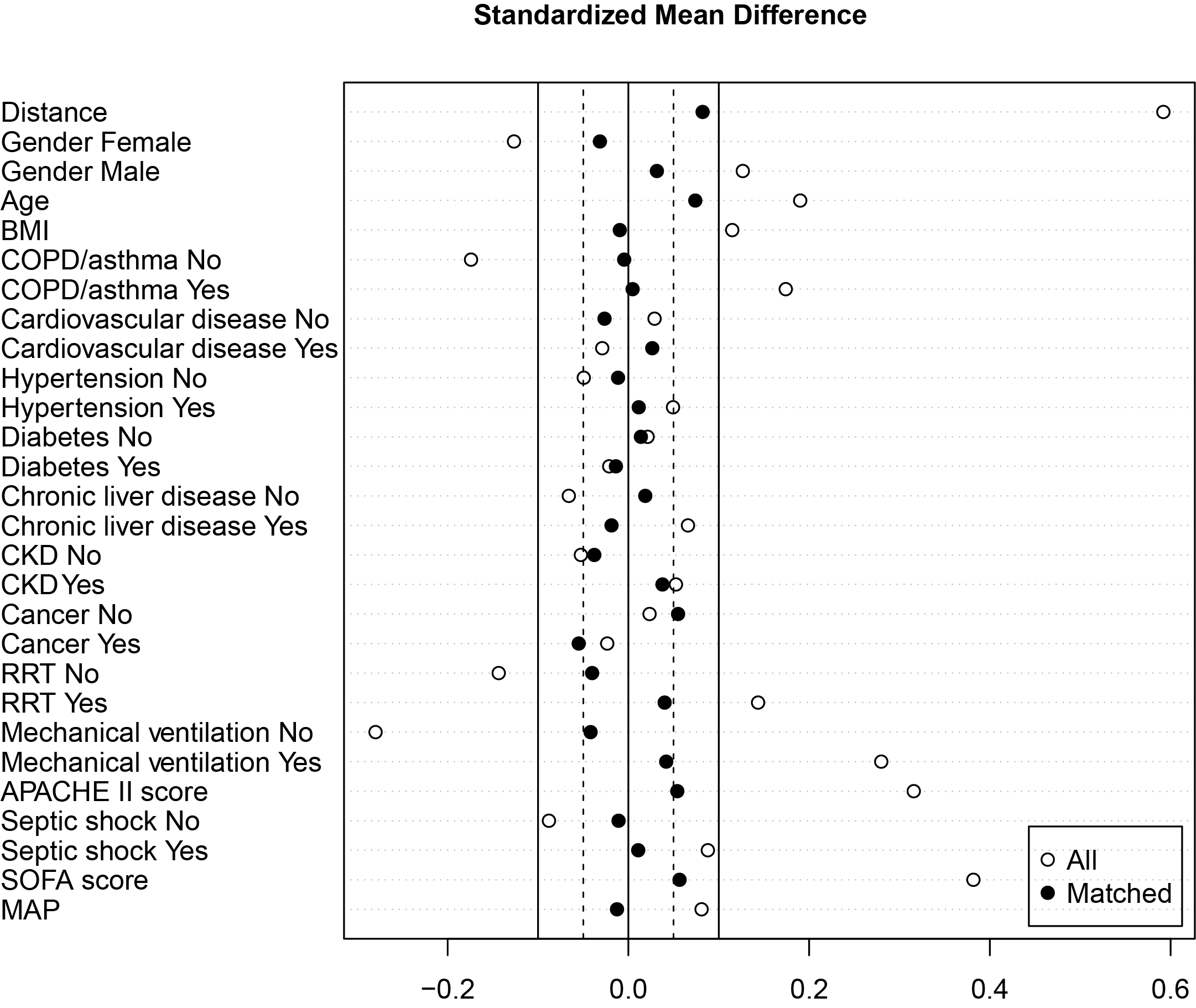


**Supplemental Material Fig. S4. The standardized difference before and after matching in cohorts with and without ARDS**

Abbreviations: ARDS, acute respiratory distress syndrome; BMI, body mass index; COPD, chronic obstructive pulmonary disease; CKD, chronic kidney disease; RRT, renal replacement therapy; APACHE II, acute physiologic and chronic health evaluation II; SOFA, sequential organ failure assessment; MAP, mean arterial pressure

**Supplemental Material Table S3. Results of the sensitivity analysis of 30-day attributable mortality**

| **Gamma** | **Lower bound** | **Upper bound** |
| --- | --- | --- |
| 1 | 0 | 0.00000 |
| 2 | 0 | 0.00000 |
| 3 | 0 | 0.00063 |
| 4 | 0 | 0.41436 |
| 5 | 0 | 0.97739 |
| 6 | 0 | 0.99991 |

**Supplemental Material Table S4. The optimal multivariable Cox proportional hazard regression analysis for 30-day mortality stratified by severity of ARDS**

| **Variables** | **HR (95% CI)** | ***p-*value** |
| --- | --- | --- |
| ARDS Severity | | |
| Mild ARDS | 1.30 (1.03-1.64) | 0.027 |
| Moderate ARDS | 1.49 (1.20-1.85) | <0.001 |
| Severe ARDS | 1.95 (1.51-2.52) | <0.001 |
| Age | 1.03 (1.02-1.03) | <0.001 |
| Cancer | 1.41 (1.11-1.78) | 0.005 |
| RRT | 1.48 (1.24-1.77) | <0.001 |
| APACHE II score | 1.05 (1.04-1.07) | <0.001 |
| MAP | 0.99 (0.99-1.00) | <0.001 |

Abbreviations: ARDS, acute respiratory distress syndrome; RRT, renal replacement therapy; APACHEII, acute physiologic and chronic health evaluation II; MAP, mean arterial pressure

**Supplemental Material Table S5. The full model of the multivariable Cox regression**

| **Variables** | **HR (95%CI)** | ***p-*value** |
| --- | --- | --- |
| ARDS Severity | | |
| Mild ARDS | 1.31 (1.03-1.65) | 0.026 |
| Moderate ARDS | 1.51 (1.21-1.88) | <0.001 |
| Severe ARDS | 1.99 (1.53-2.59) | <0.001 |
| Male | 1.02 (0.86-1.20) | 0.843 |
| Age | 1.03 (1.02-1.03) | <0.001 |
| BMI | 0.99 (0.97-1.01) | 0.449 |
| Chronic comorbidities | | |
| COPD/asthma | 0.95 (0.73-1.23) | 0.688 |
| Cardiovascular disease | 1.06 (0.87-1.30) | 0.562 |
| Hypertension | 0.99 (0.83-1.18) | 0.907 |
| Diabetes | 1.04 (0.86-1.26) | 0.689 |
| Chronic liver disease | 1.50 (0.91-2.48) | 0.112 |
| Cancer | 1.39 (1.09-1.76) | 0.008 |
| CKD | 0.80 (0.61-1.07) | 0.130 |
| RRT | 1.52 (1.26-1.84) | <0.001 |
| Mechanical ventilation | 0.91 (0.73-1.15) | 0.434 |
| APACHE II score | 1.05 (1.04-1.07) | <0.001 |
| Septic shock | 1.08 (0.88-1.33) | 0.444 |
| SOFA score | 1.00 (0.98-1.03) | 0.876 |
| MAP | 0.99 (0.99-1.00) | 0.027 |

Abbreviations: ARDS, acute respiratory distress syndrome; BMI, body mass index; COPD, chronic obstructive pulmonary disease; CKD, chronic kidney disease; RRT, renal replacement therapy; APACHE II, acute physiologic and chronic health evaluation II; SOFA, sequential organ failure assessment; MAP, mean arterial pressure

**
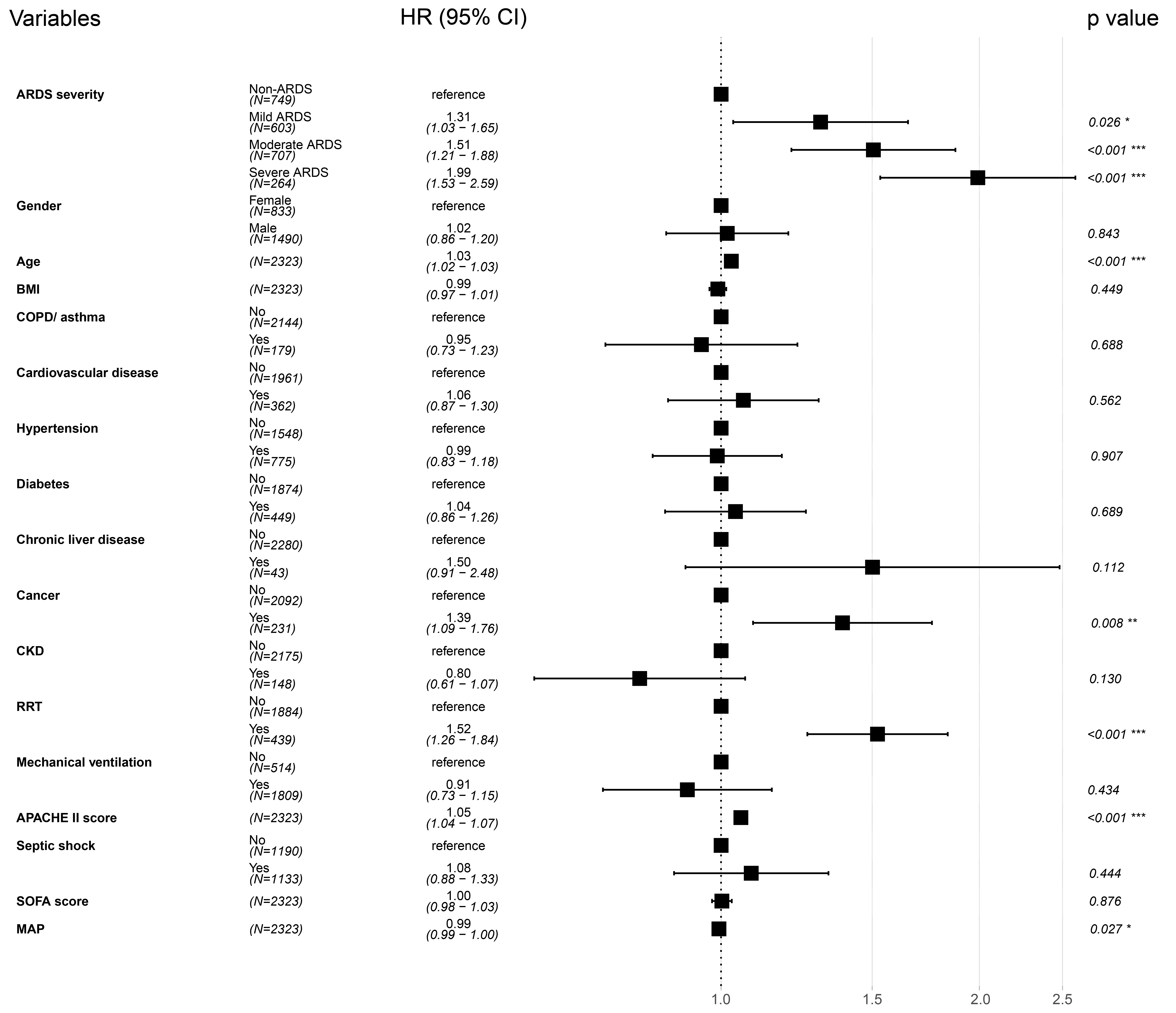
**

**Supplemental Material Fig. S5. The forest plot for full model of the multivariable Cox regression**

Abbreviations: ARDS, acute respiratory distress syndrome; BMI, body mass index; COPD, chronic obstructive pulmonary disease; CKD, chronic kidney disease; RRT, renal replacement therapy; APACHE II, acute physiologic and chronic health evaluation II; SOFA, sequential organ failure assessment; MAP, mean arterial pressure
